# *Olindias deigo* sp. nov., a new species (Hydrozoa, Trachylinae, Limnomedusae) from the Ryukyu Archipelago, southern Japan

**DOI:** 10.3897/zookeys.900.38850

**Published:** 2019-12-31

**Authors:** Sho Toshino, Miyako Tanimoto, Ryo Minemizu

**Affiliations:** 1 Kuroshio Biological Research Foundation, 560 Nishidomari, Otsuki, Kochi 788-0333, Japan Kuroshino Biological Research Foundation Nishidomari Japan; 2 Okinawa Churaumi Aquarium, 424 Ishikawa, Motobu, Okinawa 905-0206, Japan Okinawa Churaumi Aquarium Motobu Japan; 3 Ryo Minemizu Photo Office, 224-1, Yahata, Shimizu, Sunto, Shizuoka 411-0906, Japan Ryo Minemizu Photo Office Shimizu Japan

**Keywords:** Development, flower hat jellyfish, hydroid, medusa, Okinawa, polyp

## Abstract

A new hydromedusa belonging to the order Limnomedusae is reported from the Ryukyu Archipelago, southern Japan. *Olindias deigo***sp. nov.** can be distinguished from other Olindiidae species by the number and color of tentacles. Mature medusae of *O. deigo***sp. nov.** were collected to observe the life history, including polyp (hydroid) and medusa formation. A comparative table of the primary diagnostic characters of the genus is provided.

## Introduction

The order Limnomedusae comprises about 60 species in five families: Armorhydridae Swedmark & Teissier, 1958; Geryoniidae Eschscholtz, 1829; Microhydrulidae Bouillon & Deroux, 1967; Monobrachiidae Mereschkowsky, 1877; and Olindiidae Haeckel, 1879 (Bouillon et al. 2006; Bentlage et al. 2018). Olindiidae is the largest family which includes 16 genera and 49 species (Bentlage et al. 2018). The species of Olindiidae have been reported from the Pacific and the Atlantic in tropical, subtropical, mild, and cold localities (Mayer 1910; Kramp 1961). Most species inhabit salt waters; however, some species have been found in fresh and brackish waters (Oka and Hara 1922; Jankowski 2001; Toyokawa and Fujii 2015). Olindiidae species have a planktonic sexual medusa and a benthic asexual polyp in their life cycles (Kakinuma 1971; Nagao 1973; Toshino 2017; Kayashima et al. 2019).

Species of the genus *Olindias* Müller, 1861 are large hydrozoans with umbrella diameters reaching 10 cm (Kramp 1961). *Olindias formosus* (Goto, 1903) is a very beautiful jellyfish called the “flower hat jellyfish” and is popular for exhibition in public aquaria worldwide (Yasuda 2003; Patry et al. 2014). Venomous stings by another species, *Olindias sambaquiensis* Müller, 1861, have been reported around South American seashores, and it is regarded as a venomous jellyfish (Mianzan and Ramírez 1996; Resgalla et al. 2011). To date, a single incidence of lethal envenomation has been documented for *O. formosus* in Japan (Yasuda 1988; Purcell et al. 2007), which occurs near seashore.

Recently, ten specimens of *Olindias* were collected from Okinawa Island, southern Japan. In this study, morphology and molecular phylogenetic analyses revealed that the specimens represent a new species of *Olindias*. Furthermore, we observed and documented the life history of this new species of *Olindias*.

## Material and methods

### Collection and fixing

**Figure 1.**
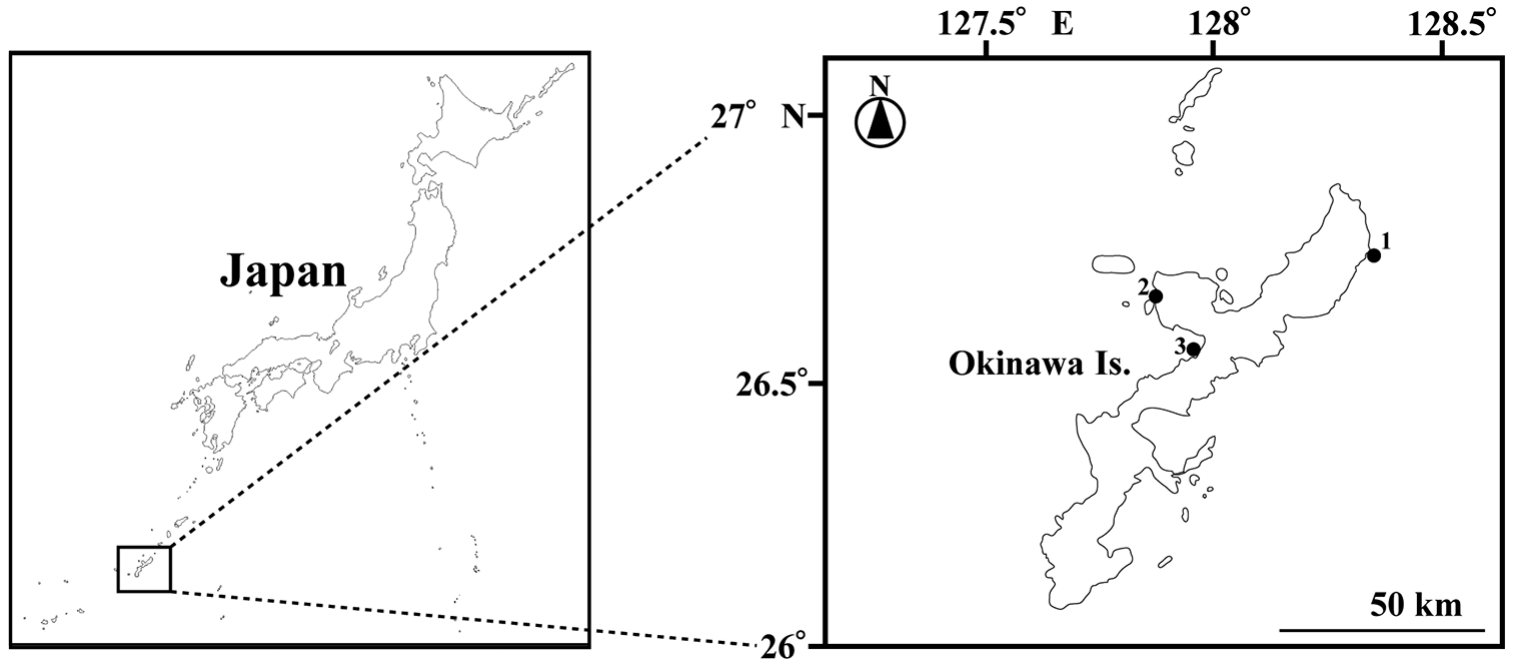
Map of the sampling sites **1** Off Ada, Kunigami **2** Off Motobu, Motobu **3** Off Kyoda, Nago.

Ten medusae were collected from Kunigami, Motobu, and Nago, Okinawa Prefecture, Ryukyu Archipelago, southern Japan between March 29, 2015 and April 8, 2018 (Fig. 1). The medusae were collected using a dipper net (diameter 20 cm) and plastic bags while scuba diving, or a set net. Additionally, specimens of *O. formosus* collected from Iwate, Oita and Miyazaki prefectures were used for comparison of morphology and for molecular phylogenetic analyses (Table 1). After preserving a subsample in ethanol (for molecular analysis), collected medusae were fixed in 5% formalin seawater and deposited in the National Museum of Nature and Science, Tsukuba, Japan (**NSMT**). Part of the tentacles were preserved in 99.5% ethanol for DNA extraction.

### Morphological investigations

**Figure 2.**
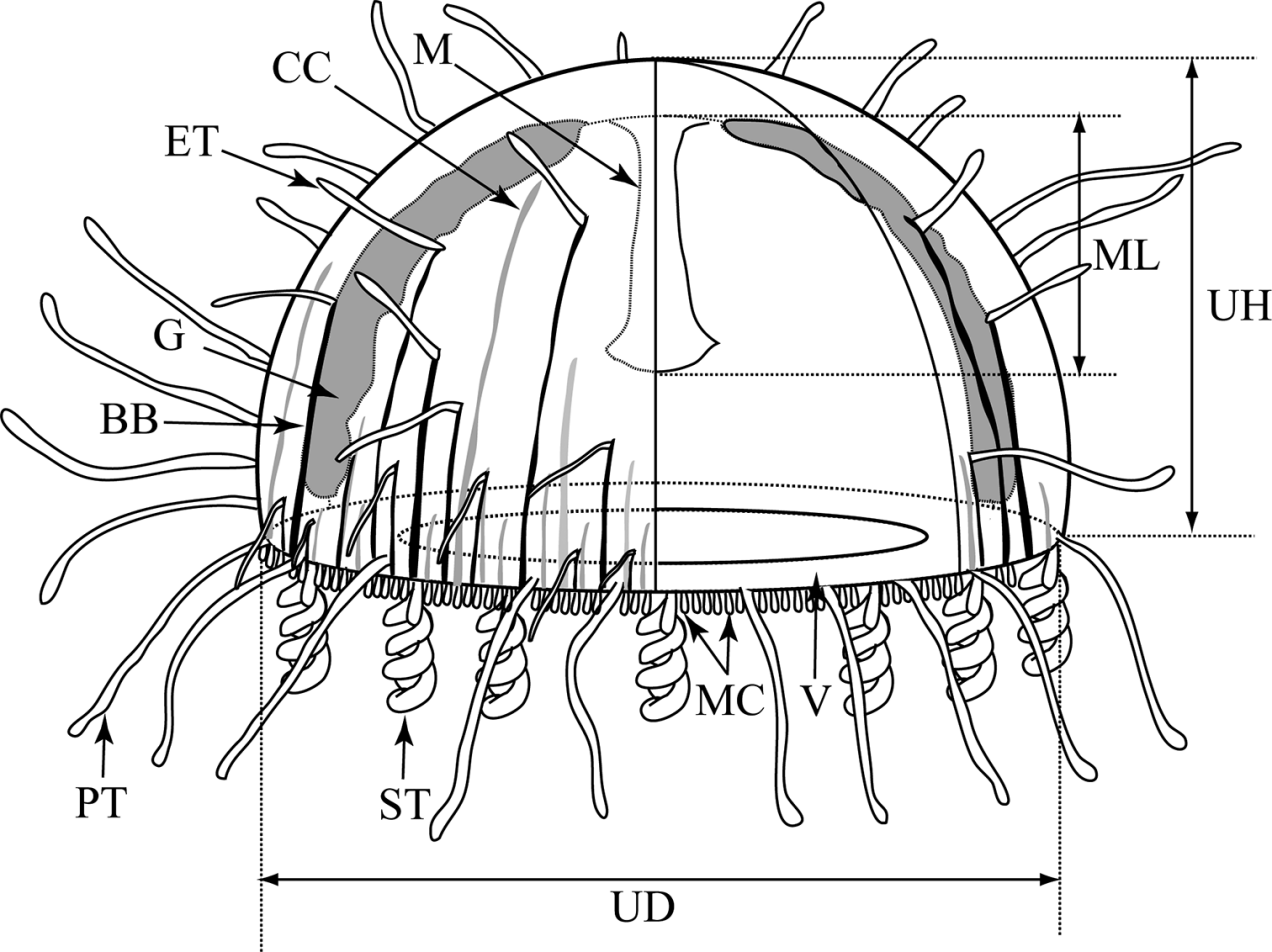
Key characters for identification and measurement of parts of the *Olindias*. BB = Black band; CC = centripetal canal; ET = exumbrella tentacle; G = gonad; M = manubrium; MC = marginal club; ML = manubrium length; PT = primary tentacle; ST = secondary tentacle; UH = umbrella height; UD = umbrella diameter; V = velum.

Morphological observations and measurements were made on living or preserved specimens. Measurements were made with digital calipers (CD-20CPX, Mitutoyo Corporation, Japan) to the nearest 0.01 mm. For nematocyst identification in the medusae, squashes prepared from fresh tissues were examined under a compound microscope (BX53, OLYMPUS, Japan). In this study, the following morphological characters were recorded (Fig. 2): umbrella height, umbrella diameter, number of centripetal canals, primary tentacles, secondary tentacles, marginal clubs, and exumbrella tentacles. Goto (1903) distinguished exumbrella tentacles, those arising from the exumbrella at any level, from those occurring proximal to the apex – just a short distance from the velum; however, he did not distinguish exumbrella from primary tentacles. In this study, the exumbrella tentacles are defined as tentacles that arise from the black band on the exumbrella, rather than those arising from the margin of the umbrella.

Nematocysts were identified according to Östman (2000) from wild and cultured specimens. Measurements were made using ImageJ (NIH, USA) to the nearest 0.1 µm.

### Molecular phylogenetic analyses

Near complete sequences of the nuclear 16S rDNA genes (approximately 600 bp) were used for molecular phylogenetic analyses. Genomic DNA was extracted from the 99.5% ethanol preserved tissue of specimens using the DNeasy Blood and Tissue Kit (QIAGEN, Germany) following the manufacturer’s protocol. 16S rDNA was PCR amplified and sequenced using primers and protocols outlined in Collins et al. (2008). Unidentified and deposited olindiid sequences in GenBank (Table 1) were used for molecular comparison and to clarify the unidentified *Olindias* species. The generated sequences were aligned using MEGA 6.06 with built in ClustalW (Tamura et al. 2013). Phylogenetic analysis and pairwise distance measurements were determined using the maximum likelihood method with 1000 bootstrap replications in MEGA 6.06 (Tamura et al. 2013). All sequences have been deposited in DNA Data Bank of Japan (DDBJ) under accession numbers LC508961–LC508970 for the new species (Table 1).

**Table 1.** Taxa included in the phylogenetic analyses and accession numbers for sequences. Sequences obtained in this study are in bold. a Collins et al. (2008); b Collins et al. (2005); c He et al. unpublished; d Goto et al. unpublished; e Patry et al. unpublished; f Bentlage et al. (2018).

**Species**	**Accession No.**	**Locality (Origin)**	**Reference**
*Aglauropsis aeora*	EU293973	Unknown	a
*Astrohydra japonica*	EU293975	Universität Hamburg, Germany	a
*Craspedacusta sinensis*	AY512507	China	b
*Craspedacusta sowerbyi*	EU293971	Unknown	a
*Craspedacusta ziguiensis*	EU293974	Unknown	a
*Gonionemus* sp.	KF962480	Unknown	c
*Gonionemus vertens*	EU293976	Friday Harbor, WA, USA	a
*Limnocnida tanganyicae*	EU293972	Unknown	a
*Maeotias marginata*	AY512508	Suisun Bay, CA, USA	a
*Monobrachium parasiticum*	EU293970	Unknown	a
*Scolionema suvaense*	AB720909	Unknown	d
***Olindias deigo***	**LC508961**	Ada, Kunigami, Okinawa, Japan	This study
***Olindias deigo***	**LC508962**	Ada, Kunigami, Okinawa, Japan	This study
***Olindias deigo***	**LC508963**	Motobu, Okinawa, Japan	This study
***Olindias deigo***	**LC508964**	Kyoda, Nago, Okinawa, Japan	This study
***Olindias formosus***	**LC508965**	Nagoya, Saiki, Oita, Japan	This study
***Olindias formosus***	**LC508966**	Nobeoka, Miyazaki, Japan	This study
***Olindias formosus***	**LC508967**	Nobeoka, Miyazaki, Japan	This study
***Olindias formosus***	**LC508968**	Nobeoka, Miyazaki, Japan	This study
***Olindias formosus***	**LC508969**	Nobeoka, Miyazaki, Japan	This study
***Olindias formosus***	**LC508970**	Ryori Bay, Ofunato, Iwate, Japan	This study
*Olindias formosus*	KF184031	Unknown	e
*Olindias mulleri* (identified as *O. phosphorica*)	AY512509	Mallorca	b
	EU293978	Unknown	a
*Olindias sambaquiensis*	EU293977	Brazil	a
*Olindias tenuis*	MG979369	Atrantic	f

### Observation of life cycle

Collected male and female medusae were transferred to an aquarium tank (18 × 32 × 22 cm, volume 13 L) to obtain fertilized eggs. Spawning was induced by alternation of light and dark conditions using an LED lamp (8 W) with a blue filter. The medusae were incubated in light between 20:30 and 7:00 and in dark between 7:00 and 20:30. Obtained fertilized eggs were kept in Petri-dishes (diameter 8 cm, height 4 cm) with filtered seawater (5 μm) at about 20 °C in the laboratory at Okinawa Churaumi Aquarium. *Artemia* nauplii were fed to primary and secondary polyps twice to thrice a week. Full water changes were conducted with filtered seawater (5 μm) about three hours after feeding. Newly detached medusae were kept in Petri-dishes (diameter 8 cm, height 4 cm) with filtered seawater (5 μm) at about 20 °C. *Artemia* nauplii were fed to the young medusae daily. The medusae that grew to about 4 cm of umbrella diameter were transferred into a tank (38 × 48 × 58 cm, volume 96 L). Juvenile anchovies and krill were fed to the medusae daily. Culture water was replaced with filtered seawater (5 μm) about three hours after feeding.

## Results

### Phylum Cnidaria Verrill, 1865

**Subphylum Medusozoa Peterson, 1979**

**Class Hydrozoa Owen, 1843**

**Subclass Trachylinae Haeckel, 1879**

**Order Limnomedusae Kramp, 1938**

**Family Olindiidae Haeckel, 1879**

**Genus *Olindias* Müller, 1861**

#### Olindias deigo sp. nov.

Taxon classificationAnimaliaLimnomedusaeOlindiidae

FA358507-CCDE-5E71-A8F9-7FEA4BDFB13F

http://zoobank.org/84DCB028-70AE-4625-93F0-0A6BFB404933

Figs 3 , 4 , 5 , 6 , 7 , 8 , 9 , 10

##### New Japanese name.

Deigo-hanagasa-kurage.

##### Material examined.

***Holotype***: NSMT-Co1690. Ada, Kunigami, Okinawa Prefecture, Ryukyu Archipelago, southern Japan; 26°43'29.0"N, 128°19'7.0"E; March 11, 2018; collector: Shuhei Odoriba. ***Paratypes***: NSMT-Co1691. Same locality as holotype, March 16, 2018, collector: Shuhei Odoriba. NSMT-Co1692. Motobu, Okinawa Prefecture, Ryukyu Archipelago, southern Japan; 26°40'18.0"N, 127°52'49.0"E; April 19, 2015; collector: Shinichi Arakawa.

##### Description.

**Figure 3.**
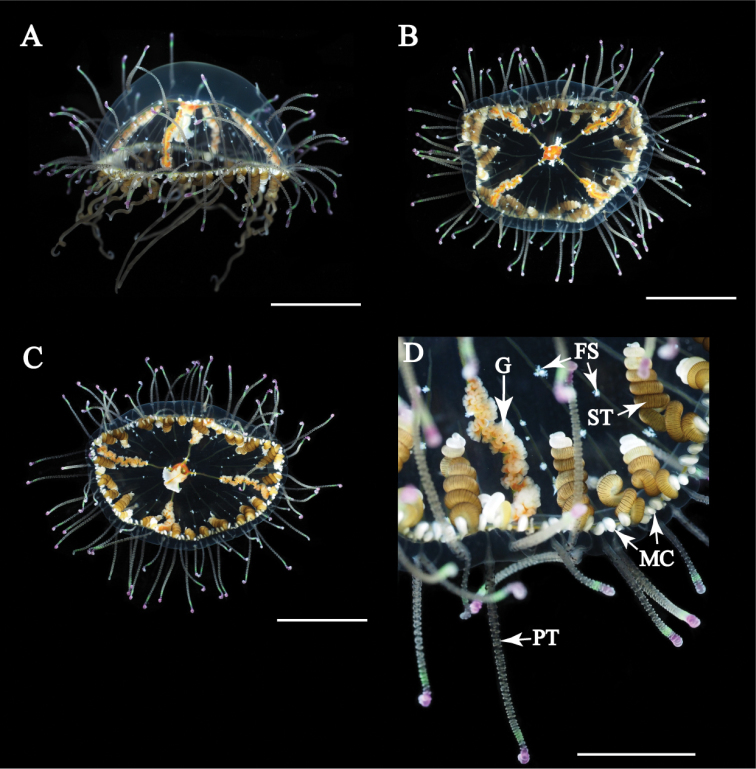
*Olindias deigo* sp. nov., live **A** lateral view **B** apical view **C** oral view **D** umbrella margin. FS = fibrous structure; G = gonad; MC = marginal club; PT = primary tentacle; ST = secondary tentacle. Scale bars: 2 cm (**A–C**), 1 cm (**D**).

**Figure 4.**
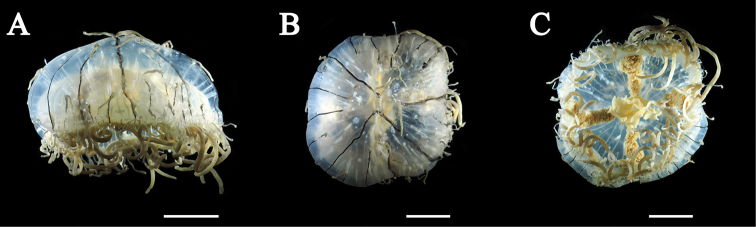
*Olindias deigo* sp. nov., holotype **A** lateral view **B** apical view **C** oral view. All scale bars represent 2 cm.

**Figure 5.**
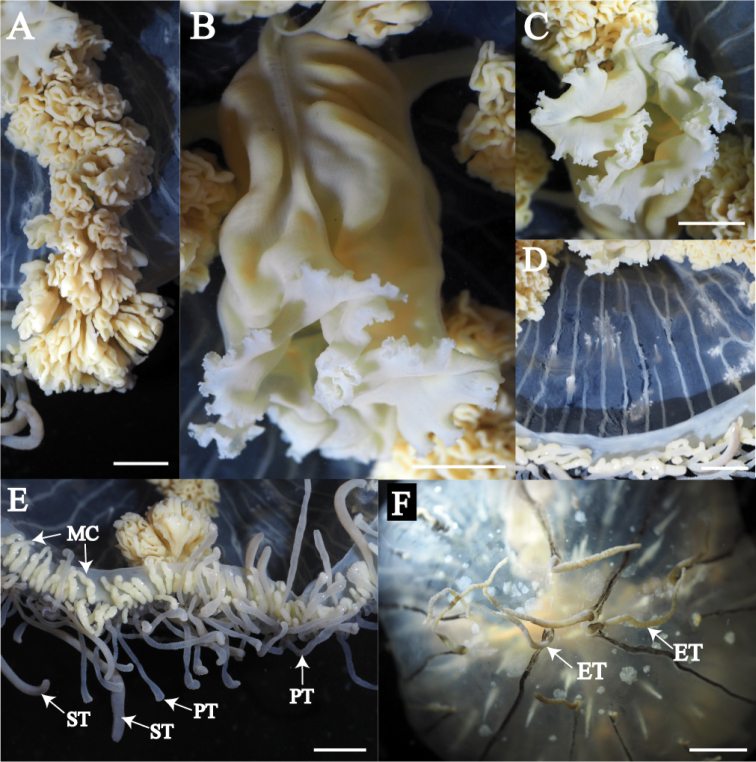
*Olindias deigo* sp. nov., holotype **A** gonad **B** manubrium **C** mouth rips **D** centripetal canals **E** umbrella margin **F** exumbrella. ET = exumbrella tentacle; MC = marginal club; PT = primary tentacle; ST = secondary tentacle. Scale bars: 0.5 cm (**A–E**), 1 cm (**F**).

Mature medusae with transparent, dome-like exumbrella (Figs 3A, 4A). Umbrella height about 40 mm and umbrella diameter about 80 mm (Table 2). Exumbrella smooth, lacking nematocyst warts (Fig. 3B). Four radial canals elongate from four corners of stomach (Figs 3B, C, 4B). Folded gonads attached along entire length of four radial canals (Fig. 5A). Immature gonads light red to orange (Figs 3D, 4C) while mature gonads are milky-white in color. Manubrium long, length about 50% of umbrella height, with quadrate base, light red to orange in color, folded (Fig. 5B, C). Mouth quadrate to rhomboid (Fig. 5C). Oral rips complexly folded (Fig. 5C). White fibrous structures scattered in mesoglea of exumbrella (Fig. 5D). Different length of black bands elongated from umbrella margin to the apex of exumbrella (Fig. 5F). Centripetal canals about 80 to 100, long and short alternately aligned (Fig. 5D). Long canals reached apex of the umbrella while short ones were half or quarter length that of long canals terminating in tentacles. Some canals connected or branched (Fig. 5D). Tentacles and marginal clubs aligned on the umbrella margin (Figs 3D, 5E). Primary tentacles about 80 to 140, thin, short with distal adhesive pads and cnidocysts in transverse clasps. Color of exumbrella tentacles and primary tentacles pale black with purple and glowing green tips and with black base (Fig. 3D). Number of secondary tentacles about 50, thick, no adhesive pads, cnidocysts in rings, deep-brown in color (Fig. 3D). Contracted secondary tentacle short, coil-like while elongate ones reaching 2 m in length. Exumbrella tentacles about 30 to 60, developing on tip of black bands (Fig. 5F). Shape and color similar to those of primary tentacles (Fig. 3D). Number of marginal clubs about 170 to 240, rounded, short, whitish in color (Fig. 3D). Statocysts were not found in fixed mature medusae.

**Table 2.** Size (mm) of *Olindias deigo* sp. nov. *: the holotype. Nos. Co1691-1692 are paratypes. **: damaged. CC = centripetal canal; ET = exumbrella tentacle; PT = primary tentacle; MC = Marginal club; ST = secondary tentacle; UD = umbrella width; UH = umbrella height.

**Specimen No.**	**UH (mm)**	**UD (mm)**	**No. of ET**	**No. of CC**	**No. of PT**	**No. of ST**	**No. of MC**	**Sampling site**	**Date**	**Lat./ long.**
NSMT-Co1690*	39.5	67.1	33	83	112	51	238	Ada, Kunigami, Okinawa	11/03/2018	26°43'29.0"N, 128°19'7.0"E
NSMT-Co1691	44.7	83.7	66	104	141	(29)**	242	Ada, Kunigami, Okinawa	16/03/2018	26°43'29.0"N, 128°19'7.0"E
NSMT-Co1692	29.9	61.8	30	86	78	49	168	Motobu, Okinawa	19/04/2015	26°40'18.0"N, 127°52'49.0"E

##### Life cycle.

**Figure 6.**
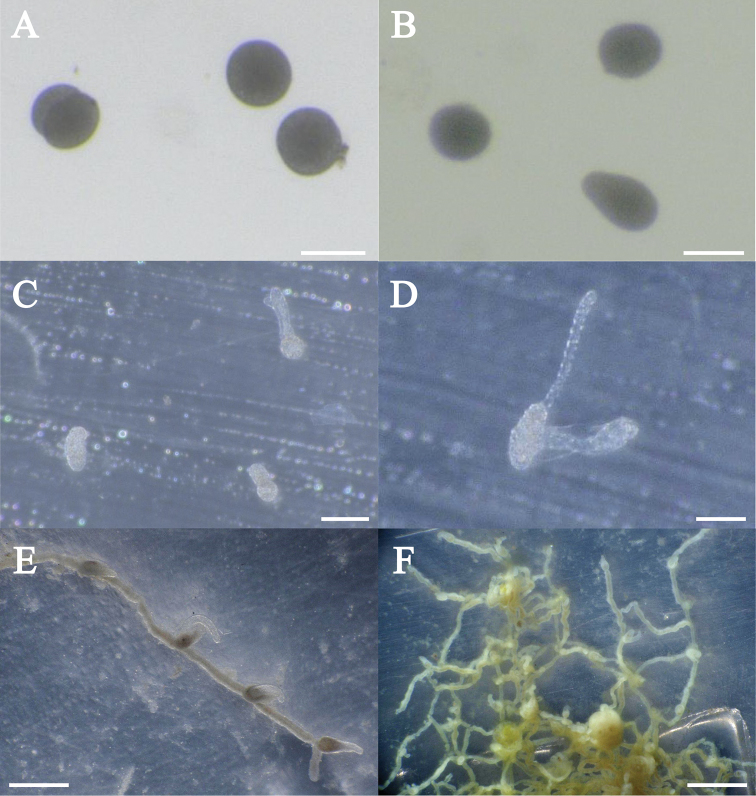
Early embryogenesis and polyps of *Olindias deigo* sp. nov. **A** fertilized eggs **B** planulae **C–D** primary polyps **E–F** mature polyps. Scale bars: 0.1 mm (**A–B**), 0.2 mm (**C–D**), 1 mm (**E–F**).

***Fertilization and polyp formation*.** Spawning occurred in dark conditions. Thousands of fertilized eggs were collected from the bottom of the tank in the early morning (from 8 to 9 am); diameter of blastocysts ~100 µm (Fig. 6A). Blastocysts developed into planulae within two days. Planulae had a pear-shaped body, 70 µm in diameter and 130 µm in length (Fig. 6B); they developed into polyps within 20 days.

The polyps form small colonies by elongation of the stolon, developing into a network (Fig. 6C–F). The hydrorhizae were cylindrical with small egg-shaped or cylindrical hydranths developing on the stolon. The hydranths had an ovoid body, 0.7 mm in length (Fig. 6E). The body was divided in two parts, gastric region (0.3 mm in diameter and 0.5 mm in length) and hypostome (0.2 mm in diameter and 0.2 mm in length). Tentacle single, filiform, 1.7 mm in length (Fig. 6E, F). The hydroid, usually brownish or yellowish, became orange or pink owing to the consumption of *Artemia* nauplii. Tentacle and hypostome transparent.

**Figure 7.**
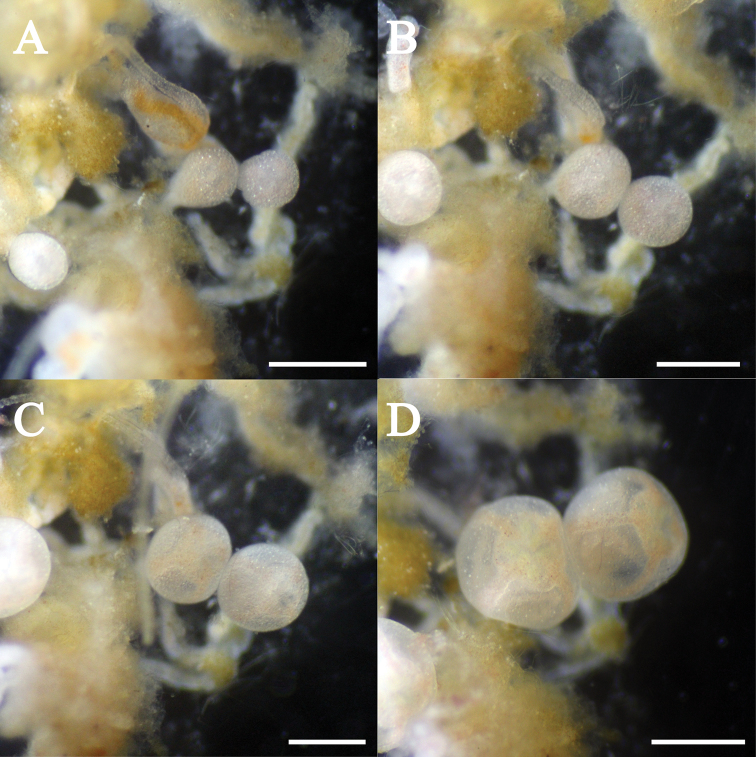
The process of medusa budding in the hydroid of *Olindias deigo* sp. nov. All scale bars represent 1 mm.

***Budding and development of young medusa*.** Budding of young medusae was observed after 8 months of polyp formation. Medusa bud formation occurred on stolon (Fig. 7A) at temperatures below 20 °C. The shape of the buds was ovoid and 0.3 mm in diameter (Fig. 7A). Two days after onset of budding, four radial canals and a circular canal appeared, but were obscure (Fig. 7B). Eight days after onset of budding, rudiments of tentacles developed from the bud (Fig. 7C). Fourteen days after onset of budding, the buds enlarged (0.8 mm in diameter) and green fluorescence was observed on the tentacles (Fig. 7D). Fifteen days after onset of budding, the medusa buds detached.

**Figure 8.**
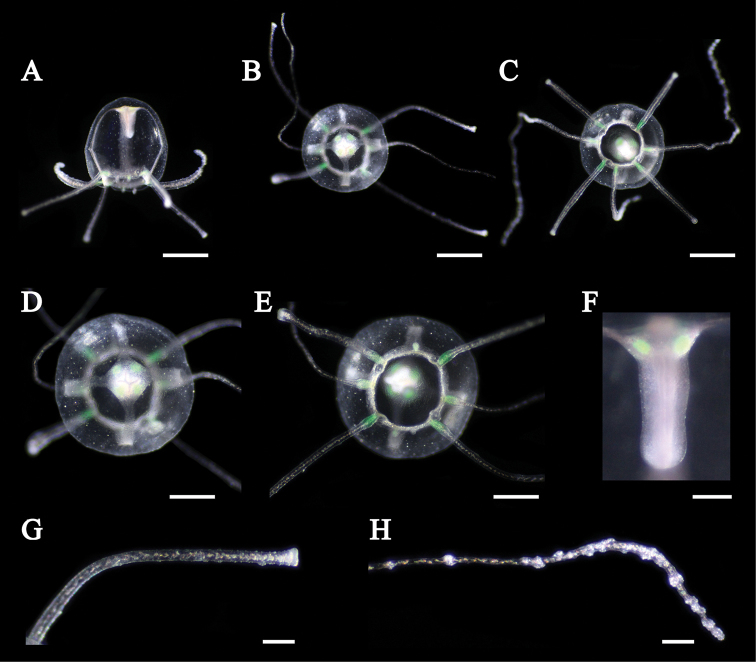
Newly detached medusa of *Olindias deigo* sp. nov. **A** lateral view **B, D** apical view **C, E** oral view **F** manubrium **G** primary tentacle **H** secondary tentacle. Scale bars: 1 mm (**A–C**), 0.5 mm (**D, E**), 0.1 mm (**F–H**).

Newly detached medusae had a spherical umbrella translucent in color (Fig. 8A–C). Umbrella height about 1.6 mm and diameter about 1.5 mm. Exumbrella with tiny nematocysts along entire exumbrella (Fig. 8D). Four simple radial canals from four corners of the stomach (Fig. 8B, D). Statocysts four, enclosed in mesoglea, adjacent to primary tentacles (Fig. 8E). Manubrium long, about 50% that of umbrella height (Fig. 8F). Marginal tentacles of two types (Fig. 8C, G, H). Primary tentacles four, short (about 1 to 2 times that of umbrella diameter) bearing nematocyst clusters on the tips (Fig. 8G). Secondary tentacles two, long (about 5 times that of umbrella diameter) bearing 10 to 20 nematocyst batteries (Fig. 8H). The medusae attached using the tip of the primary tentacles, but adhesive pad was not observed (Fig. 8G). Green fluorescence was observed at the base of tentacles and four corners of the stomach (Fig. 8D–F).

**Figure 9.**
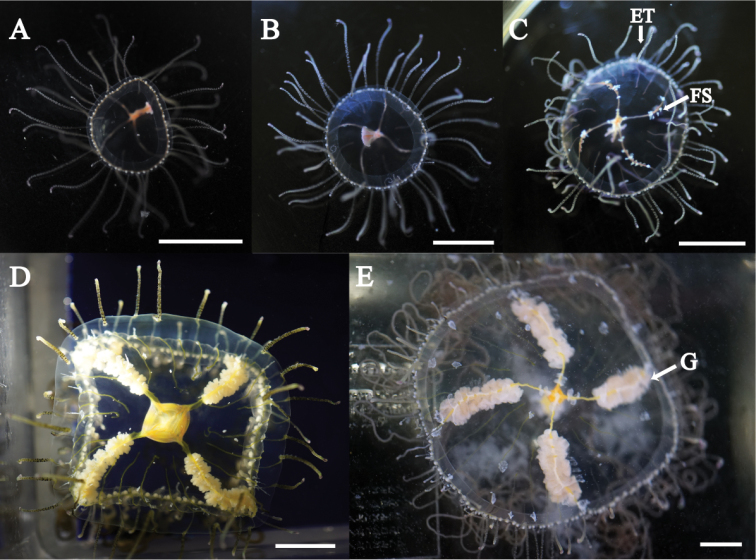
The process of young medusa development in *Olindias deigo* sp. nov. ET = exumbrella tentacle; FS = fibrous structure; G = gonad. All scale bars represent 1 cm.

Ninety-day-old medusae were about 10 mm in diameter (Fig. 9A). Umbrella bowl-shaped. Primary and secondary tentacles about 40 and 20, respectively. About 20 centripetal canals were observed. Medusae aged 120-day-old were about 15 mm in diameter (Fig. 9B). White fibrous structures appeared around radial canals. Manubrium elongated and mouth rips developed. Number of primary and secondary tentacles and radial canals not increased much. Medusae aged 150-day-old were about 20 mm in diameter (Fig. 9C). Primary and secondary tentacles about 60 and 20, respectively. About 20 centripetal canals observed. Exumbrella tentacles developed near umbrella margin, but were not observed on the apex of exumbrella. Medusae aged 200-day-old were about 40 mm in diameter (Fig. 9D). Primary and secondary tentacles about 80 and 40, respectively. About 60 centripetal canals were observed. Gonad developed. Exumbrella tentacles developed near the margin of umbrella and the middle part of exumbrella. Medusae aged 240-day-old were about 60 mm in diameter (Fig. 9E). Primary and secondary tentacles about 120 and 40, respectively. About 60 centripetal canals observed. Gonad developed and matured. Spawning observed (Fig. 9E).

**Figure 10.**
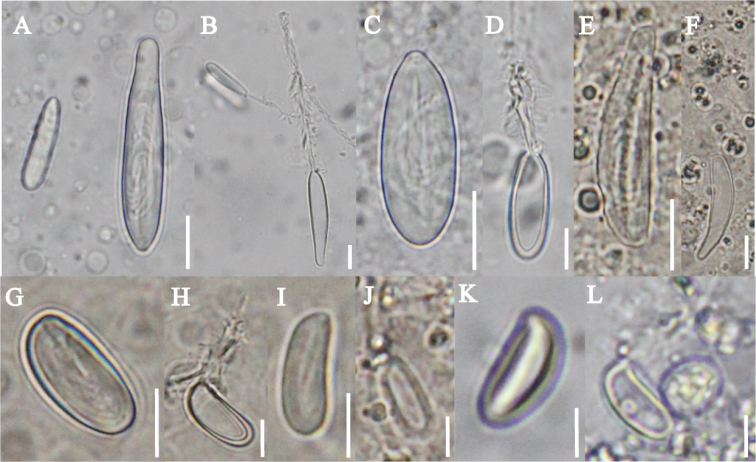
Nematocysts of *Olindias deigo* sp. nov. **A, B** macrobasic b-mastigophore (small and large), adult medusae. Intact (**A**), discharged (**B**) **C, D** eurytele, adult medusae. Intact (**C**), discharged (**D**) **E, F** macrobasic b-mastigophore, young medusae. Intact (**E**), discharged (**F**) **G, H** eurytele (Large), young medusae. Intact (**G**), discharged (**H**) **I, J** eurytele (Small), young medusae. Intact (**I**), discharged (**J**) **K, L** microbasic eurytele, mature polyp. Intact (**K**), discharged (**L**). Scale bars: 10 µm (**A–F**), 5 µm (**G–L**).

***Cnidome.*** Two different nematocyst types were identified and measured in the adult medusae, young medusae, and mature polyps (Table 3). Adult medusae had two nematocyst types. Two sizes of macrobasic b-mastigophores (Fig. 10A, B) and microbasic euryteles (Fig. 10C, D) were found on primary, secondary, and exumbrella tentacles. Young medusae had two nematocyst types. Macrobasic b-mastigophores (Fig. 10E, F) were found only on tentacles while two sizes of microbasic euryteles (Fig. 10G–J) were found on primary, secondary, and exumbrella tentacles. The mature polyps had one nematocyst type, microbasic euryteles (Fig. 10K, L).

**Table 3.** Cnidomes of *Olindias deigo* sp. nov. D, L represent capsule diameter and length, respectively, in μm.

**Stage**	**Part**	**Type**		**Min**	**Max**	**Mean**	**SD**	**N**
Adult medusae	Primary tentacle	Macrobasic p-mastigophore (Large)	D	5.69	8.75	7.37	0.63	50
			L	34.19	42.44	38.95	1.99	50
		Macrobasic p-mastigophore (Small)	D	3.24	5.15	4.02	0.45	50
			L	13.01	18.58	16.48	1.18	50
		Microbasic eurytele	D	8.01	10.91	9.84	0.77	50
			L	20.56	28.48	24.61	1.94	50
Young medusae	Exumbrella	Microbasic eurytele (Large)	D	5.66	8.32	7.10	0.72	14
			L	13.70	20.02	17.62	1.78	14
		Microbasic eurytele (Small)	D	2.09	4.68	3.40	0.49	28
			L	6.39	10.47	8.64	1.07	28
	Tentacle	Macrobasic p-mastigophore	D	6.04	7.85	6.77	0.46	50
			L	26.29	34.62	30.42	2.25	50
		Microbasic eurytele (Large)	D	6.33	9.49	7.70	0.68	44
			L	15.70	23.62	20.04	2.35	44
		Microbasic eurytele (Small)	D	2.62	4.33	3.53	0.43	50
			L	6.82	11.97	9.37	1.03	50
Hydroids	Body	Microbasic eurytele	D	4.01	8.31	5.59	0.72	100
			L	9.29	16.95	12.62	1.50	100
	Tentacle	Microbasic eurytele	D	3.79	7.35	5.93	0.72	94
			L	10.75	16.61	13.05	1.20	94

##### Molecular phylogenetics.

**Figure 11.**
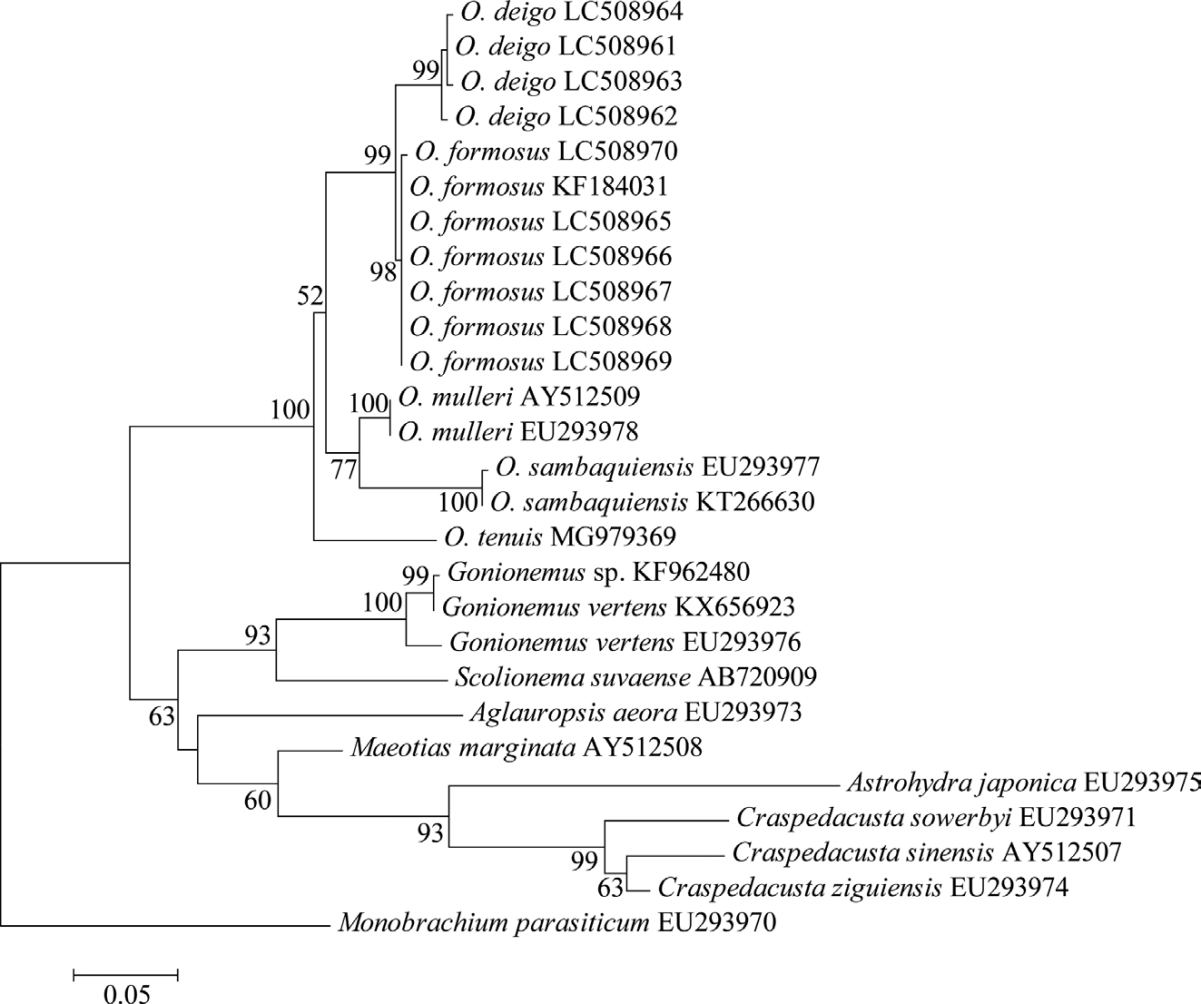
Maximum likelihood tree for 15 limnomedusan taxa based on the nuclear 16S rDNA data set. Scale bars indicate branch length in substitutions per site. Nodal support values are presented as the ML bootstrap value; only values >50% are shown.

In the resulting maximum likelihood tree (Fig. 11), four major monophyletic clades were formed in the genus *Olindias*: 1) *O. formosus*; 2) *Olindias muelleri* Haeckel, 1879; 3) *O. sambaquiensis*; 4) *Olindias tenuis* (Fewkes, 1882); and 5) a fifth group (*O. deigo*). The monophyly of *O. deigo* was evident in the 16S phylogenetic tree with high bootstrap values (99%), strongly supporting the validity of the new species. The Kimura 2-parameter distance between *O. deigo* and *O. formosus* was 0.03, below the distance 0.06–0.11 between olindiids (Table 4). Interspecific distance 0.000–0.002 between *O. formosus* from Iwate Prefecture, eastern Japan and *O. formosus* from Oita and Miyazaki prefectures, western Japan. Therefore, K2P divergence factor between 0.03–0.11 could be a threshold for discriminating olindiid species.

**Table 4.** Pairwise genetic distances (K2P) based on 410 positions of 16S sequences among Limnomedusae. The analysis involved 27 sequences.

No.		1	2	3	4	5	6	7	8	9	10	11	12	13	14	15	16	17	18	19	20	21	22	23	24	25	26
1	*Aglauropsis aeora* EU293973																										
2	*Astrohydra japonica* EU293975	0.226																									
3	*Craspedacusta sinensis* AY512507	0.230	0.220																								
4	*Craspedacusta sowerbyi* EU293971	0.258	0.197	0.089																							
5	*Craspedacusta ziguiensis* EU293974	0.220	0.194	0.051	0.073																						
6	*Gonionemus* sp. KF962480	0.178	0.236	0.229	0.247	0.210																					
7	*Gonionemus vertens* EU293976	0.187	0.246	0.239	0.253	0.216	0.030																				
8	*Gonionemus vertens* KX656923	0.178	0.233	0.226	0.243	0.206	0.002	0.027																			
9	*Maeotias marginata* AY512508	0.145	0.203	0.175	0.183	0.151	0.154	0.160	0.154																		
10	*Scolionema suvaense* AB720909	0.201	0.243	0.213	0.233	0.191	0.133	0.130	0.130	0.169																	
11	*Olindias deigo* LC508961	0.198	0.263	0.237	0.240	0.213	0.207	0.200	0.203	0.188	0.178																
12	*Olindias deigo* LC508962	0.201	0.263	0.237	0.237	0.207	0.207	0.200	0.203	0.188	0.178	0.005															
13	*Olindias deigo* LC508963	0.204	0.263	0.233	0.233	0.204	0.203	0.197	0.200	0.184	0.175	0.007	0.002														
14	*Olindias deigo* LC508964	0.204	0.267	0.240	0.240	0.210	0.210	0.197	0.207	0.191	0.181	0.007	0.002	0.005													
15	*Olindias formosus* LC508965	0.188	0.253	0.230	0.237	0.204	0.200	0.187	0.197	0.169	0.181	0.027	0.027	0.025	0.030												
16	*Olindias formosus* LC508966	0.188	0.253	0.230	0.237	0.204	0.200	0.187	0.197	0.169	0.181	0.027	0.027	0.025	0.030	0.000											
17	*Olindias formosus* LC508967	0.188	0.253	0.230	0.237	0.204	0.200	0.187	0.197	0.169	0.181	0.027	0.027	0.025	0.030	0.000	0.000										
18	*Olindias formosus* LC508968	0.189	0.254	0.230	0.237	0.204	0.201	0.188	0.197	0.169	0.182	0.028	0.028	0.025	0.030	0.000	0.000	0.000									
19	*Olindias formosus* LC508969	0.189	0.254	0.230	0.237	0.204	0.201	0.188	0.197	0.169	0.182	0.028	0.028	0.025	0.030	0.000	0.000	0.000	0.000								
20	*Olindias formosus* LC508970	0.188	0.253	0.233	0.240	0.207	0.200	0.187	0.197	0.169	0.181	0.030	0.030	0.027	0.033	0.002	0.002	0.002	0.002	0.002							
21	*Olindias formosus* KF184031	0.188	0.253	0.230	0.237	0.204	0.200	0.187	0.197	0.169	0.181	0.027	0.027	0.025	0.030	0.000	0.000	0.000	0.000	0.000	0.002						
22	*Olindias mulleri* AY512509	0.217	0.274	0.244	0.254	0.213	0.210	0.197	0.206	0.175	0.191	0.072	0.072	0.069	0.074	0.061	0.061	0.061	0.061	0.061	0.064	0.061					
23	*Olindias mulleri* EU293978	0.217	0.274	0.244	0.254	0.213	0.210	0.197	0.206	0.175	0.191	0.072	0.072	0.069	0.074	0.061	0.061	0.061	0.061	0.061	0.064	0.061	0.000				
24	*Olindias sambaquiensis* EU293977	0.214	0.260	0.253	0.257	0.226	0.220	0.216	0.220	0.197	0.204	0.094	0.096	0.094	0.099	0.088	0.088	0.088	0.088	0.088	0.088	0.088	0.066	0.066			
25	*Olindias sambaquiensis* KT266630	0.218	0.263	0.257	0.260	0.229	0.216	0.213	0.216	0.194	0.200	0.096	0.099	0.096	0.102	0.091	0.091	0.091	0.091	0.091	0.091	0.091	0.064	0.064	0.002		
26	*Olindias tenuis* MG979369	0.217	0.253	0.233	0.230	0.213	0.207	0.207	0.207	0.157	0.200	0.096	0.096	0.093	0.099	0.088	0.088	0.088	0.088	0.088	0.091	0.088	0.077	0.077	0.108	0.105	
27	*Monobrachium parasiticum* EU293970	0.234	0.344	0.314	0.351	0.299	0.265	0.258	0.261	0.218	0.276	0.241	0.241	0.241	0.245	0.224	0.224	0.224	0.225	0.225	0.224	0.224	0.231	0.231	0.259	0.262	0.262

##### Habitat and ecology.

Medusae of *O. deigo* appeared in shallow waters (from 3 to 10 m) during winter and spring in a range of subtropical temperature localities in the Ryukyu Archipelago, southern Japan. The medusae rested on the sandy bottom or in areas with a good slope and movement of water during the daytime while they drifted and swam by extending their tentacles during the night. Thus, the species seems to be nocturnal in behavior. Stinging events attributable to *O. deigo* have not been reported thus far.

##### Etymology.

The species name comes from the beautiful appearance of the jellyfish. The Japanese name *deigo* (noun in apposition) means *Erythrina variegata* which is popular as the “prefectural flower” of Okinawa.

##### Differential diagnosis.

A comparison of key features of the species in the genus *Olindias* is presented in Table 5. All species of *Olindias* have four radial canals and numerous centripetal canals; numerous tentacles of two kinds: primary ones issuing above the umbrella margin, with distal adhesive pads and cnidocysts in transverse clasps and secondary ones on the umbrella margin, no adhesive pads, cnidocyst in rings; gonads with papilliform processes, on radial canals; numerous marginal clubs, statocyst usually in pairs at base of primary tentacles (Bouillon et al. 2006). *Olindias deigo* can be distinguished from other Olindiidae species by the number and color of tentacles in adult medusae. Many more primary tentacles than secondary tentacles in *O. deigo*, *O. formosus*, and *O. singularis*, while fewer primary tentacles than secondary tentacles in *O. malayensis*, *O. muelleri*, *O. sambaquiensis*, and *O. tenuis* (Table 5). Several exumbrella tentacles present in *O. deigo* and *O. formosus* while lacking in others. Exumbrella tentacles of *O. deigo* many more than those of *O. formosus* (84 vs 30–60, respectively). The primary tentacles were colorful (black, purple, and glow green) in *O. deigo* and *O. formosus*, while they were red and yellow in *O. malayensis*, *O. muelleri*, *O. sambaquiensis*, and *O. tenuis* (no data for *O. singularis* and *Olindias* sp.) (Table 5).

**Table 5.** Morphology of adult medusae in previous and the present study. Bars represent a lack of data.

	*O. deigo* sp. nov.	*O. formosus*		*O. malayensis*	*O. mulleri*		*O. sambaquiensis*	*O. singularis*	*O. tenuis*	*Olindias* sp. (young medusa)
UD (mm)	62–84	83.2	75	25–35	40–60	22–44	50–100	13–36	35	7
UH (mm)	30–45	42.6	about 1/2 of UD	over 1/2 of UD	–	–	–	half of UD	–	5.5
No. of ET	30–60	84	present	absent	absent	absent	absent	absent	absent	absent
No. of PT	78–141	168	264	20–30	50–60	48–60	80–100	28–86	32–54	12
No. of ST	49–51	57	325	30–40	100–120	96–120	200–300	16–50	38–70	–
No. of MC	168–242	283	–	120	100–170	–	100–200	32 to more than 100	64–69	–
No. of CC (per quadrant)	20–26	19–23	18–23	7–9	11–19	7–11	21–27	4–12	7–10	1
No. of gonads	4	4–6	4–6	4	4	4	4	4	4	4
Gonads	Folded/ along nearly whole length of radial canals	Folded/ along nearly whole length of radial canals	–	Papilliform/ along nearly whole length of radial canals	Linear, swollen, with surfaces covered with branched processes/ over nearly entire length of radial canals	Papilliform/along the radial canals	Folded/ along nearly whole length of radial canals	Papilliform/ outer half of radial canals	Papilliform/ outer half of radial canals	Folded/ upper half of the radial canals
Statocysts	Not examined	Not examined	Twice as many as primary tentacles	Twice as many as primary tentacles	Twice as many as primary tentacles	–	Twice as many as primary tentacles	Single otolith at base of each primary tentacle	Single otolith at base of each tentacle	Two at the base of two centripetal canals
Color	Manubrium light red to orange. Gonads milky-white. Primary tentacles pale black with purple and glowing green tips and black base. Secondary tentacles deep-brown.	Manubrium lilac to red orange. Each corner of base of manubrium smaragdine-green. Gonads egg-yellow. Tips of primary and exumbrellar tentacles transparent, lilac and smaragdine-green. Marginal clubs and base of primary and exumbrella tentacles ivory-black. Radial canals and circular canals deep scarlet. Centripetal canals lighter scarlet.	–	Similar to *O. mulleri*	Similar to *O. tenuis* but apparently browner and duller.	Gonads orange	Bright and variable, with mingled yellow, red, brown, and black. Colors similar to *O. tenuis*.	Entoderm of stomach, gonads, and ring-canal opaque (cream color?).	Entoderm of manubrium, tentacle-bulbs, and gonads opaque yellowish-green, streaked with purple. Exumbrella tentacles white or dark-purple. Marginal tentacles red and yellow.	–
Distribution (Sampling locality)	Ryukyu archipelagos, Okinawa, southern Japan	Oita, Japan	Japan; Korea	Malay Archipelago	Bahamas; Bermudas; Mediterranean Sea; West Africa	Aegean Sea	Brazil; Argentina	Maldive Is.; Low Archipelago; Chagos Archipelago; Philippines; India Australia; Iranian Gulf; Pakistan	Florida; Bahamas; Barmudas; Cuba	Sunda Strait
References	This study	This study	Goto (1903) Kramp (1961) Park (2006)	Maas (1905) Mayer (1910) Kramp (1961)	Mayer (1910) Kramp (1961)	Aytan et al. (2019)	Müller (1861) Mayer (1910) Kramp (1961) Chiaverano et al. (2004)	Browne (1905) Mayer (1910) Kramp (1961)	Fewkes (1883) Maas (1905) Mayer (1910) Kramp (1961)	Uchida (1947)

## Discussion and conclusions

Prior to our study, only one olindiid, *O. formosus*, had been recorded from Japan (Goto 1903). This species was described by Goto (1903) based on specimens collected from Misaki, Kanagawa Prefecture, eastern Japan. The medusae of the species have been reported from warm and cold localities in the Sea of Japan and the Pacific coast of Honshu (Uchida and Uchida 1965), and Jejudo Island, Korea (Park 2006). Distribution of the two species, *O. formosus* and *O. deigo*, do not overlap.

Development of olindiids is known in only two species *O. formosus* (Patry et al. 2014) and *O. muelleri* (identified as *Olindias phosphorica* (Delle Chiaje, 1841)) (Weill 1936). Polyps form colonies which are stolonal, and hydroids bear a single tentacled hydranth, but lack hydrotheca in *O. deigo* and *O. formosus* (Patry et al., 2014) (Table 5). However, polyps of *O. muelleri* are solitary, and hydranth lacks tentacle but enclosed by hydrotheca. Young medusae of *O. deigo* resemble those of *O. formosus* in umbrella sizes and number of tentacles (Patry et al. 2014).

Asexual reproduction and medusa budding of *O. deigo* were observed at 20 °C. The temperature corresponds with that of winter waters around Okinawa Island (Japan Meteorological Agency 2019). Mature medusae appear between winter and spring in Okinawa. Polyps of *O. deigo* may produce medusae during fall and winter.

Morphological and molecular phylogenetic analyses in this study provide evidence that *Olindias* from the Ryukyu Archipelago is a new species. Olindiids are very beautiful and popular but harmful because of their venomous stings (Mianzan and Ramírez 1996; Resgalla et al. 2011). Additional investigations are needed to understand the ecology and distribution of *O. deigo*.

## Supplementary Material

XML Treatment for Olindias deigo

## References

[B1] Aytan Ü Aksu İ Bektaş Y (2019) Recent occurrence of *Olindias muelleri* Haeckel, 1879 (Cnidaria, Hydrozoa, Limnomedusae, Olindiidae) in the Aegean Sea. Plankton and Benthos Research 14(1): 22–28. 10.3800/pbr.14.22

[B2] Bentlage B Osborn KJ Lindsay DJ Hopcroft RR Raskoff KA Collins AG (2018) Loss of metagenesis and evolution of a parasitic life style in a group of open-ocean jellyfish. Molecular phylogenetics and evolution 124: 50–59. 10.1016/j.ympev.2018.02.030 29518561

[B3] Bouillon J Gravili C Gili JM Boero F (2006) An introduction to Hydrozoa. Mémoires du Muséum National d’Histoire Naturelle 194: 1–591.

[B4] Browne ET (1905) Report on the medusae (Hydromedusae, Scyphomedusae and Ctenophora) collected by Prof. Herdman at Ceylon in 1902. Report to the Government of Ceylon on Pearl Oyster Fisheries of the Gulf of Manaar 4: 132–166.

[B5] Chiaverano L Mianzan H Ramírez F (2004) Gonad development and somatic growth patterns of *Olindias sambaquiensis* (Limnomedusae, Olindiidae). Hydrobiologia 530(1–3): 373–381. 10.1007/s10750-004-2666-4

[B6] Collins AG Winkelmann S Hadrys H Schirwater B (2005) Phylogeny of Capitata and Corynidae (Cnidaria, Hydrozoa) in light of mitochondrial 16S rDNA data. Zoologica Scripta 34: 91–99. 10.1111/j.1463-6409.2005.00172.x

[B7] Collins AG Bentlage B Lindner A Lindsay D Haddock SHD Jarms G Norenburg JL Jankowski T Cartwright P (2008) Phylogenetics of Trachylina (Cnidaria: Hydrozoa) with new insights on the evolution of some problematical taxa. Journal of the Marine Biological Association of the United Kingdom 88: 1673–1685. 10.1017/S0025315408001732

[B8] Fewkes JW (1883) On a few medusae from the Bermudas, in Exploration of the surface fauna of the Gulf Stream, under the auspices of the United States Coast Survey, by Alexander Agassiz. Bulletin of the Museum of Comparative Zoology at Harvard University 11(3): 79–90.

[B9] Goto S (1903) The craspedote medusa *Olindias* and some of its natural allies. Mark Anniversary 1: 1–22. 10.5962/bhl.title.3959

[B10] Jankowski T (2001) The freshwater medusae of the world – a taxonomic and systematic literature study with some remarks of other inland water jellyfish. Hydrobiologia, 462: 91–113. 10.1023/A:1013126015171

[B11] Japan Meteorological Agency: Climate Statistics. Normals (1981–2010) . http://www.data.jma.go.jp/obd/stats/data/en/normal/normal.html [accessed on 1 November 2019]

[B12] Kakinuma Y (1971) Life cycle of a hydrozoan, *Gonionema oshoro* Uchida. The bulletin of the Marine Biological Station of Asamushi, Tohoku University 14: 91–97.

[B13] Kayashima H Tanabe S Kakihara Y Ishii H (2019) Effects of temperature on the reproduction type of *Scolionema suvaense* living on seaweed and seagrass. Plankton and Benthos Research 14(2): 55–61. 10.3800/pbr.14.55

[B14] Kramp PL (1961) Synopsis of the medusae of the world. Journal of the Marine Biological Association of the United Kingdom 40: 1–469. 10.1017/S0025315400007347

[B15] Maas O (1905) Die craspedoten medusen der Siboga-expedition. Siboga Expeditie 10: 1–84. 10.5962/bhl.title.11301

[B16] Mayer AG (1910) Medusae of the world. Carnegie Institution of Washington 109: 1–735. 10.5962/bhl.title.159245

[B17] Mianzan HW Ramírez FC (1996) *Olindias sambaquiensis* stings in the South West Atlantic. In: Williamson JAH Fenner PJ Burnett JW Rifkin JF (Eds) Venomous and Poisonous Marine Animals: a Medical and Biological Handbook.University of New South Wales Press, Brisbane, 206–208.

[B18] Müller OF (1861) Polypen und quallen von Santa Catharina. *Olindias sambaquiensis*, n. sp. Archiv für Naturgeschichte 27: 312–319.

[B19] Nagao Z (1973) The life history of *Eperetmus typus* Bigelow and the systematics of the family Olindiadidae (Limnomedusae). Publications of the Seto Marine Biological Laboratory 20: 89–102. 10.5134/175787

[B20] Oka A Hara M (1922) On a new species of *Limnocodium* from Japan. Annotationes zoologicae japonenses 10: 83–87.

[B21] Östman C (2000) A guideline to nematocyst nomenclature and classification, and some notes on the systematic value of nematocysts. Scientia Marina 64 (Supplement 1): 31–46. 10.3989/scimar.2000.64s131

[B22] Park JH (2006) New Records of Some Hydromedusae (Cnidaria: Hydrozoa) in Korea. Animal Systematics, Evolution and Diversity 22(2): 169–177.

[B23] Patry W Knowles T Christianson L Howard M (2014) The hydroid and early medusa stage of *Olindias formosus* (Cnidaria, Hydrozoa, Limnomedusae). Journal of the Marine Biological Association of the United Kingdom 94(7): 1409–1415. 10.1017/S0025315414000691

[B24] Purcell JE Uye SI Lo WT (2007) Anthropogenic causes of jellyfish blooms and their direct consequences for humans: a review. Marine Ecology Progress Series 350: 153–174. 10.3354/meps07093

[B25] Resgalla Jr C Rosseto AL Haddad Jr V (2011) Report of an outbreak of stings caused by *Olindias sambaquiensis* Müller, 1861 (Cnidaria: Hydrozoa) in southern Brazil. Brazilian Journal of Oceanography 59(4): 391–396. 10.1590/S1679-87592011000400009

[B26] Tamura K Stecher G Peterson D Filipski A Kumar S (2013) MEGA6: Molecular Evolutionary Genetics Analysis version 6.0. Molecular Biology and Evolution 30: 2725–2729. 10.1093/molbev/mst197 24132122 PMC3840312

[B27] Toshino S (2017) *Scolionema sanshin* sp. n., a new species (Hydrozoa, Limnomedusae, Olindiidae) from the Ryukyu Archipelago, southern Japan. Zootaxa 4344(2): 277–290. 10.11646/zootaxa.4344.2.4 29245632

[B28] Toyokawa M Fujii N (2015) First record of two invasive hydromedusae *Maeotias marginata* (Modeer, 1791) (Hydrozoa: Limnomedusae) and *Blackfordia virginica* Mayer, 1910 (Hydrozoa: Leptomedusae) in Japan. Plankton and Benthos Research 10(4): 215–219. 10.3800/pbr.10.215

[B29] Uchida T (1947) Some medusae from the Central Pacific. Journal of the Faculty of Science, Hokkaido (Imperial) University, ser. VI, Zoology 7(3): 297–319.

[B30] Uchida K Uchida T (1965) New Illustrated Encyclopedia of the Fauna of Japan. Hokuryu-Kan, Tokyo, 679 pp. [in Japanese]

[B31] Weill R (1936) Existence de larves polypoïdes dans le cycle de la Trachyméduse *Olindias phosphorica* Della Chiaje. Comptes Rendus de l’Acade´mie des Sciences 203: 1018–1020.

[B32] Yasuda T (1988) Studies on the common jellyfish, *Aurelia aurita* (Linne). Japan Fisheries Resource Conservation Association, Tokyo, 136 pp. [in Japanese with English abstract]

[B33] Yasuda T (2003) Jellyfish: UFO of the sea. Kouseishakouseikaku, Tokyo, 206 pp. [in Japanese]

